# Recurrent Metastatic Chordoma to the Liver: A Case Report and Review of the Literature

**DOI:** 10.3390/curroncol29070367

**Published:** 2022-06-30

**Authors:** Thomas E. Dickerson, Asad Ullah, Sathvik Saineni, Sandresh Sultan, Srikar Sama, Intisar Ghleilib, Nikhil G. Patel, Islam A. Elhelf, Nagla Abdel Karim

**Affiliations:** 1 Medical College of Georgia, Augusta University, Augusta, GA 30912, USA; thdickerson@augusta.edu (T.E.D.); drsaineni@gmail.com (S.S.); srikar.sama@gmail.com (S.S.); 2 Department of Pathology, Augusta University, Augusta, GA 30912, USA; aullah@augusta.edu (A.U.); ighleilib@augusta.edu (I.G.); npatel4@augusta.edu (N.G.P.); 3 Department of Medicine, Bolan Medical College, Quetta 83700, Pakistan; sandreshsultan89@gmail.com; 4 Department of Radiology, Augusta University, Augusta, GA 30912, USA; ielhelf@augusta.edu; 5 Inova Schar Cancer Institute, University of Virginia, Fairfax, VA 22031, USA

**Keywords:** malignant, chordoma, magnetic resonance imaging

## Abstract

Chordoma is a rare malignant neoplasm derived from notochordal tissue that primarily affects the axial skeleton. Almost 40% of patients have non-cranial chordoma metastases. The most common metastatic sites are the lungs, bones, lymph nodes, and subcutaneous tissue. We present a 52-year female with a history of sacral chordoma presenting with abdominal fullness, early satiety, and a palpable abdominal mass. Abdominal magnetic resonance imaging (MRI) revealed an isolated, highly vascularized, and multilobed liver mass in the left lateral segment. The mass was surgically removed using a clean surgical margin. A histological examination and immunohistochemical staining were consistent with a metastatic chordoma. Two years later, follow-up imaging studies showed a 6.5 × 4.0 × 2.0 cm right liver lesion with multiple lungs, chest wall, pleural, and diaphragmatic lesions. Microscopic- and immunohistochemical staining revealed a recurrent metastatic chordoma. Herein, we present a unique case of metastatic recurrent chordoma in the liver with the involvement of other sites. To the best of our knowledge, no other case of recurrent liver metastasis has been reported.

## 1. Introduction

Chordomas are rare primary bone tumors that originate from the notochord remnants [1]. They account for approximately 5% of malignant bone tumors and are twice as likely to occur in men [2]. Chordomas most commonly arise in the 40–60-year-old age group but can occur at any age [3]. Chordomas occur most commonly in the sacrum (50%), with the spheno-occipital region (35%) and the vertebrae (15%) being the next most common sites [4]. Chordomas are slow-growing and indolent; thus, they are often clinically silent until late in the disease, when they become locally aggressive and cause pain and neuropathy [3]. While rare, with an incidence rate of 0.08 per 100,000, a chordoma diagnosis carries a poor prognosis because of the recurrence rate, with a 67% five-year survival rate [4]. Vertebral chordomas have the worst prognosis.

Chordomas can be further subdivided into those not otherwise specified (NOS), chondroid chordoma, and dedifferentiated chordoma. Grossly, classical chordoma shows a gray-white lobulated cut surface, and microscopically, a chordoma shows epithelioid cells with an eosinophilic vacuolated cytoplasm and round nuclei with variable degrees of atypia. Chondroid chordomas show features of chordomas and chondrosarcomas. The degree of atypia associated with the tumor’s aggressiveness remains unclear [3]. Dedifferentiated chordomas are very rare; they have features of both classic chordomas with high-grade sarcoma components and a poor prognosis. Other poor prognostic factors include a large tumor size, tumor necrosis, a high Ki-67 proliferation index, and an incomplete surgical resection of the tumor [5]. High-resolution array comparative genomic hybridization has shown duplication in the 6q27 region of the T gene (brachyury), which further supports the notochord hypothesis [3]. Chordomas are diagnosed using histopathological and immunohistochemical criteria and staining for the epithelial membrane antigen (EMA), cytokeratin, and S100 [6]. There is limited research on the metastatic potential of chordomas. The metastatic rate is reported to be between 3% and 30%, with metastasis most commonly occurring in the lungs (52%). Other common metastatic sites are the bone, soft tissue, liver, lymph nodes, skin, and tongue. Local recurrence, a large tumor size, and positive surgical margins are associated with metastasis [7]. Due to the rarity of chordomas, there is currently no approved medical treatment. To date, the only curative treatment is en bloc resection with negative margins. Emerging evidence suggests that adjuvant hadron-based radiotherapy, when coupled with surgery, may be more effective in treating chordomas [6]. Unfortunately, the availability of hadron-based therapy is limited; therefore, alternatives include intensity-modulated radiation therapy (IMRT) and stereotactic delivery techniques [6]. Although a chordoma is a relatively slow-growing tumor, it is associated with a high incidence of local recurrence and with a poor long-term prognosis (8.9). The following is the case of a 52-year-old asymptomatic woman with two sequential instances of chordoma metastasis to the liver, confirmed by staining for EMA, pan-cytokeratin, vimentin, and S100. The site, its multiple recurrences, and the sex of the patient separate this case from others in the literature. A literature review found only seven other cases of metastatic disease in the liver.

Herein we report a case of recurrent metastatic chordoma on the liver after the complete resection of the primary tumor and recurrent metastatic foci from the liver.

## 2. Case Presentation

**Figure 1:**
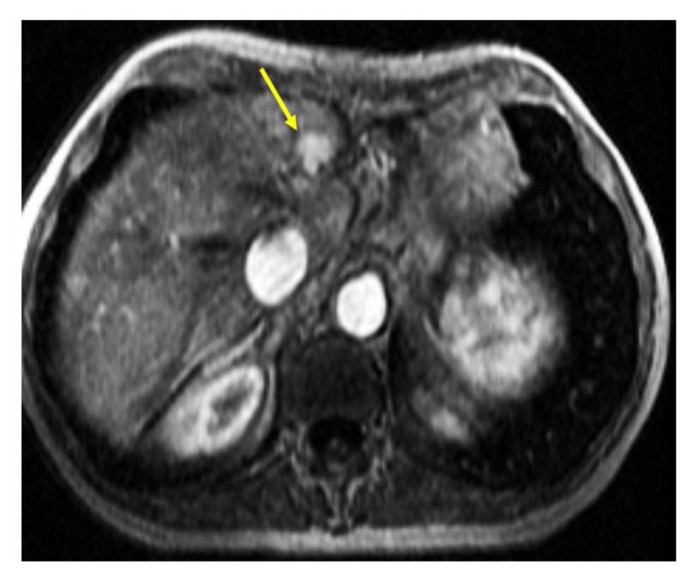
MRI of the abdomen. Contrast-enhanced MRI of the abdomen showed enhancing focal lesion in the lateral segment of the left hepatic lobe (segment 2).

A 52-year-old woman presented with a sacral mass that was excised in 1991 and was diagnosed as a chordoma at an outside institution. The surgery was complicated by the loss of bladder function and intermittent catheterization was performed. After 15 years of an asymptomatic course, she presented at our institution in December 2004 with abdominal pain and fullness, with a palpable liver mass under the left rib cage. Abdominal MRI demonstrated an isolated large mass in the left lateral segment of the left lobe of the liver. In contrast images, the mass was highly vascularized and well encapsulated (Figure 1). 

**Figure 2:**
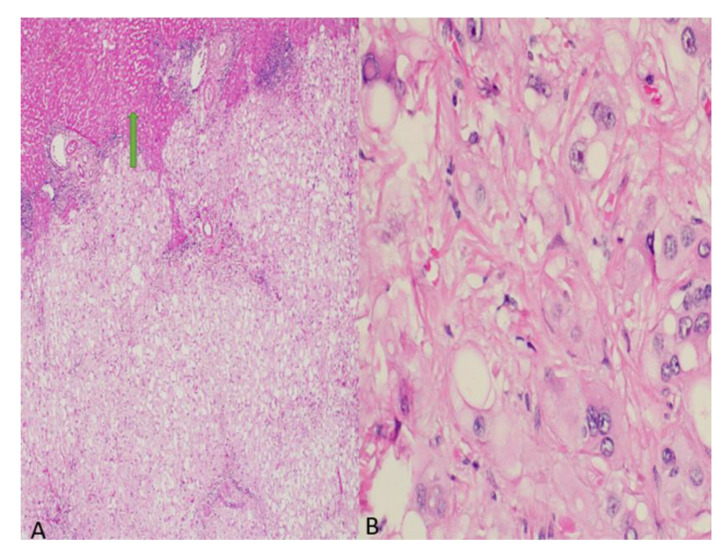
(**A**) H&E (10×): Ill-defined lesion with microcystic areas with normal liver parenchyma at the periphery (arrow). (**B**) H&E (40×): Epithelioid pleomorphic cells with prominent nucleoli with clear to eosinophilic bubbly cytoplasm.

A diagnosis of a hepatic adenoma was made based on the well-encapsulated nature of the mass and its vascularity. The mass was surgically removed using a clean surgical margin. Microscopically, the lesion revealed an infiltrative growth pattern with a lobular architecture and extracellular myxoid areas. The cells were epithelioid with abundant clear to eosinophilic cytoplasm, prominent nucleoli, and vacuolated cytoplasm (Figure 2). 

**Figure 3:**
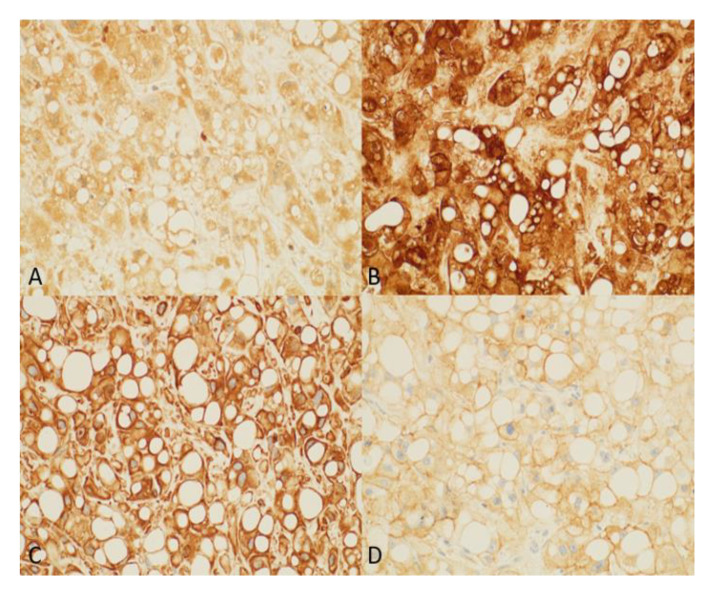
(**A**) S100 (20×): granular cytoplasmic staining of tumor cells. (**B**) EMA (20×): strong and diffuse cytoplasmic and membranous staining of the lesion. (**C**) Vimentin (20×): Diffuse staining of tumor cells cytoplasm. (**D**) EGFR (20×) stain shows membranous and cytoplasmic positivity in tumor cells.

Immunohistochemistry revealed that the tumor cells were positive for S100, vimentin, epithelial membrane antigen (EMA), and epidermal growth factor receptor (EGFR) (Figure 3). 

**Figure 4:**
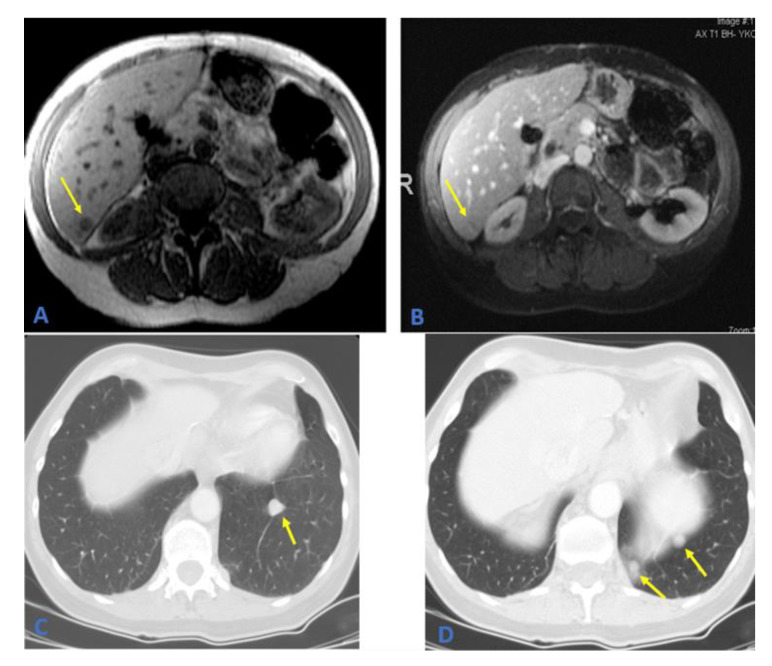
(**A**,**B**) MRI of the abdomen; T1 weighted images on the left showing hypointense hepatic focal lesion in segment 6 which is faintly enhanced on the post-contrast image on the right. (**C**,**D**) Axial CT scan of the chest showing multiple left lower lobe metastatic pulmonary nodules (arrows).

The tumor was negative for Melan A and hepatocyte-specific antigen (Hep-Par1). Based on the morphology and immunohistochemical staining, a diagnosis of a metastatic chordoma was made. Owing to her history of metastatic chordoma, the patient was scheduled to return for imaging every 6 months. The patient has been symptom-free for approximately five years. Follow-up MRI revealed a right-sided liver lesion in segment six and an enlarged left lower lobe pulmonary nodule. A complete liver ultrasound was performed, yielding a 1.2 × 1.4 cm lesion near the edge of the posterior inferior aspect of segment six (Figure 4). A further workup revealed multiple lung lesions (Figure 4). 

In the operating room, the right lobe of the liver was found to be markedly hypertrophic. A 6.5 × 4.0 × 2.0 cm mass was laparoscopically resected from segment six of the liver, in addition to a left lower lobe lung wedge resection ×6, a left chest wall resection ×2, a left anterior and posterior diaphragmatic sulcus resection, a left dome of the diaphragm resection with repair, and a left periaortic mass resection. Postsurgical changes and fibrosis in the pelvis and sacrum from the original neoplasm suggested a recurrence. The histopathological examination results were consistent with recurrent and metastatic chordomas. The incidence of a second metastatic recurrence in the liver is otherwise undescribed in the literature. The patient was scheduled to return for 3 months for a follow-up CT to check her right-sided nodules, in addition to monitoring for new left-sided lesions.

The patient returned early for repeat imaging 20 days post-op based on a new-onset fever and the presence of flank pain post resection procedure. This CT scan shows the hemoperitoneum of the right conal fascia. The hemoperitoneum was drained, and the aspirate was sent for pathology which returned negative. At the follow-up, the patient inquired about chemotherapy to prevent the recurrence of a chordoma. She was counseled and chemotherapy options were discussed. As she did not have any active tumors, she was counseled to stick to PET scan surveillance. Follow-up imaging two months later revealed new nodules in her lungs and left supraclavicular lymph nodes; however, it was decided to delay imatinib treatment as all the lesions were <1 cm. Another set of images was obtained two months later with zero progression of the nodules. The patient was lost to follow up after the imaging set.

## 3. Discussion

Chordomas are malignant tumors arising from notochordal differentiation. Three types of chordomas have been documented. Of the chordomas, 95% were the NOS (not otherwise specified) type. A Chondroid chordoma has an occurrence rate of 4%, and dedifferentiated chordoma has the lowest occurrence (<1%). The most common sites of chordomas include the clivus, sacrococcygeal bones, or vertebrae [3].

Metastatic chordomas are rare, with only 3% to 30% of cases reporting metastatic lesions [8,9]. Sacrococcygeal chordomas, such as the one reported in this patient, have shown higher rates of metastasis and recurrence than other primary site chordomas [10]. Since there are few cases of metastasis from an already rare tumor, reports of the metastatic potential of chordomas are limited. There are a few reports of metastasis to the lungs, but there are only three such reports of metastasis to the liver and no reports of recurrent metastatic lesions in the liver. The current hypothesis regarding the metastatic potential of chordomas suggests that factors such as a lack of intracellular material, variations in the cell and the nuclear size, high rates of mitosis, an increased mucin formation, pleomorphism, anaplasia, and the invasiveness of the tumor may be predictive of metastasis [11].

Small liver metastases may resemble liver cysts. A comparison with the previous images, if cystic lesions are found upon imaging in a patient with a previous chordoma, is strongly recommended [12]. Radiologically, hepatic adenomas appear hyperintense on T1 MRI and isointense on contrast. Chordomas present as round masses with a low signal intensity on T1 MRI and a honeycomb appearance with contrast [13].

Surgery is the most effective treatment for chordomas. It is usually difficult to obtain tumor-free margins during the initial surgery because the anatomical location of these tumors limits the ability of the surgeon to remove the entire tumor [14].

Chordomas have shown a poor response to chemotherapy. Various chemotherapy regimens such as anthracyclines, cisplatin, and alkylating agents, have been used in advanced chordomas but have not been effective [15].

Cetuximab and gefitinib can be considered for EGFR-positive chordomas. They both interfere with EGF signal transduction at different points. Their combination has been shown to control the disease in one case in which all other treatment options were exhausted. Their combined treatment prevented the further growth of both the primary and metastatic chordomas and even caused a slight regression over a follow-up period of 9 months. There was also an improvement in tumor-induced back pain. Cetuximab is usually well-tolerated. Acne-like skin reactions can occur and correlate with an improved treatment response. Since the drugs were administered in combination, the efficacy of each drug for chordomas must be evaluated [16].

Anti-PD-L1 therapy in metastatic chordomas is a potential therapeutic target regardless of the level of PD-L1 expression in the primary tumor. A patient with metastatic chordoma to the lungs treated with pembrolizumab showed a 30% reduction in tumor load with a PFS duration of 9.3 months. Subsequently, the tumor further metastasized to the bone. This initial response to pembrolizumab and mutations in the PBRM1 gene warrants further exploration as a potential treatment for chordomas [17].

Molecular studies have revealed the overexpression of PDGFR, KIT, and STAT3 receptors, suggesting their role as molecular targeting agents [6]. A recent study showed that the silencing of the brachyury gene resulted in the complete arrest of the growth of chordoma cells in vitro [18]. New evidence suggests that miRNA profiling will help us understand chordoma genesis and develop targeted treatments for chemo-resistant tumors [19]. The application of miRNA profiling showed that hsa-miR-31, hsa-miR-222, hsa-miR-140-3p, and hsa-miR-148a were differentially expressed in chordomas compared to healthy nucleus pulposus [20]. Zhang et al. showed that the overexpression of miR-16-5p suppresses chordoma cell proliferation, invasion, and migration. Upregulation was also correlated with the upregulated expression of E-cadherin and downregulated expression of N-cadherin [21]. Several drugs such as Palbociclib (CDK4/6 inhibitor-NCT03110744), Afatinib (TKI Inhibitor-NCT03083678), and Nivolumab (Anti PDL-1-NCT02989636) are being studied for chordoma therapy and their results are awaited [22].

Kinase inhibitors are a potential treatment for metastatic chordomas due to their heavy expression of several activated receptors. Multiple studies have shown 69–83% of chordomas to be EGFR positive, and EGFR has been shown to be the most active kinase in chordomas. Furthermore, the expression of c-MET was associated with a younger age of diagnosis and a better prognosis. PDGFR-α and EGFR were indicators of a poor prognosis [23]. A largely focused compound screen-tested 154 EGFR inhibitors against chordoma cell lines in vitro. Sapitinib, gefitinib, and erlotinib were found to be effective [24,25]. In vivo erlotinib has been successful for inhibiting the growth of chordomas [26]. Afatinib was more recently shown to have even more efficacy in chordoma treatment due to its activity against EGFR in addition to U-CH1 and UM-Chor1. The activity of Afatinib across multiple chordoma cell lines appears to promote the degradation of brachyury, drastically increasing the antitumor effects [27]. Quinoline- and quinazoline-based kinase inhibitors are active against EGFR because they affect the interaction with the site water network. 6,7-dimethoxy-N-(4-((4-methylbenzyl)oxy)phenyl) quinolin-4-amine was the most effective inhibitor of the UCH-2 chordoma cell line [28]. In 2020, a Bayesian Machine learning model was created to analyze publications of compounds tested against chordomas and generate a scoring system. The artificial intelligence (AI) found the mTOR inhibitor AZD2014 to be most potent against U-CH1 and U-CH2 expressing chordomas, while the synergistic effect of Afatinib and CDK4/6 inhibitor Palbociclib were found to be most effective in EGFR+ chordomas [29].

## 4. Conclusions

Chordomas are rare tumors with a benign course. Our research documents an unusual case of recurrent metastasis to the liver following the resection of the primary tumor and two years after liver metastasis with clean surgical margins. Although metastasis has been reported in the literature, to the best of our knowledge, our case is the first to report a recurrent liver metastasis.

## Data Availability

No new data were created or analyzed in this study. Data sharing does not apply to this article.
